# A biomechanical analysis of the roundhouse kicking technique of expert practitioners: A comparison between the martial arts disciplines of Muay Thai, Karate, and Taekwondo

**DOI:** 10.1371/journal.pone.0182645

**Published:** 2017-08-25

**Authors:** Colin J. Gavagan, Mark G. L. Sayers

**Affiliations:** School of Sport and Health Sciences, University of the Sunshine Coast, Maroochydore, Queensland, Australia; Genomics Institute of the Novartis Research Foundation, UNITED STATES

## Abstract

The purpose of this study was first, to determine whether there were differences in the roundhouse kicking leg kinematics performed by highly skilled Muay Thai, Karate and Taekwondo practitioners (n = 8 per group). Next, analysis aimed to identify the kinematic determinants of effective roundhouse kicking performance. Three-dimensional (3D) lower limb kinematics were recorded using a nine camera infra-red motion capture system (500 Hz) during three maximal roundhouse kicks. Impact forces were recorded using a strain gauge (1000 Hz) attached to a kicking pad positioned at the height of each participant’s mastoid process. Results showed that linear foot velocity at impact was moderately correlated with relative impact force (r = 0.66, P = 0.001). Discipline specific analyses of the temporal data indicated that the Muay Thai group had a shorter execution time (1.02 ± 0.15 s) than Taekwondo (1.54 ± 0.52 s, P = 0.028). Analysis of lower limb kinematic data indicated that both Karate (-947 ± 94 deg/s, P = 0.010) and Taekwondo (-943 ± 106 deg/s, P = 0.011) practitioners had faster knee extension velocities than the Muay Thai group (-706 ± 200 deg/s). Conversely, the Muay Thai practitioners (1.24 ± 0.15 m/s) had greater vertical centre of mass movement than both Karate (0.78 ± 0.24 m/s, P = 0.001) and Taekwondo groups (0.93 ± 0.19 m/s, P = 0.02). Our findings show that several fundamental movement patterns were common to the roundhouse kicking techniques across the Muay Thai, Karate, and Taekwondo disciplines. Effective roundhouse kicking performance was characterized by rapid pelvic axial rotation, hip abduction, hip flexion and knee extension velocities, combined with rapid movements of the COM towards the target.

## Introduction

The martial arts disciplines of Muay Thai, Karate, and Taekwondo evolved in relative isolation, with their evolutionary roots arising from Thailand, Japan and Korea respectively [1–5]. All three disciplines follow the same fundamental fighting and self-defence principles and are based on core skills including defensive arm and wrist locks, blocks and throws, hand and elbow striking techniques and a variety of kicks [6]. Martial artists from these disciplines commonly assume the existence of technical differences that distinguish their respective disciplines, but this concept has not been explored in the scientific literature.

A kicking technique common to all three of these martial art disciplines is the round type kick—often called the *roundhouse* or *turning kick* [7–9]. This complex kick is also the most frequently used kick in competition [10,11] and so provides and excellent tool to explore key differences, or similarities, between these popular martial arts disciplines. Another important characteristic of the roundhouse kicking technique is that it is highly adaptable, enabling practitioners to make relatively minor changes in technique to kick to target the thigh, torso, and head across a variety of distances [5].

Most biomechanical research on martial arts kicking techniques focuses on front and roundhouse kicks in Taekwondo [5,8–17]. Proportionally fewer studies report on the biomechanics of kicking techniques in Karate [18–20], with limited research available on Muay Thai [4,21]. A clear failing in this domain is that many of the biomechanical studies on martial arts kicking exist either as conference papers, are presented in an abridged form, and/or have been based on small sample sizes. Accordingly, the ability to generalise based on their findings is extremely limited. Additionally, most studies rely on two-dimensional (2D) sagittal plane analytical techniques [14,18,22,23]. Typically, studies that have reported three-dimensional (3D) kinematic data are limited to simple descriptions of joint ranges of motion (ROM) and/or segmental orientation at key phases [9,10,19,24]. These approaches have the potential to oversimplify and understate the complex movement patterns typical of all kicking activities [12,25–27]. For example, previous research on Taekwondo roundhouse kicking has suggested that the multi-planar movements of pelvis simply increase both hip ROM and kicking range [12]. Such a finding appears at odds with research on punt kicking, which shows that sagittal and transverse plane movements are critical determinants of kicking foot velocity in highly proficient kickers [26,27]. Accordingly, research that expands on the current body of knowledge and explores the role of transverse plane pelvic movements have in developing martial arts kicking force appears warranted.

O’Sullivan et al. [9] and Lee [24] are the only researchers who report kinematic data that compares roundhouse kicking techniques between martial arts styles. These researchers indicate there are little to no differences in lower limb kinematics between a traditional Taekwondo roundhouse kicking technique and that used in the more modern Martial Arts sub-disciplines of Yongmundo [9] and Hapkido [24]. The relative absence of cross-discipline biomechanical analyses on roundhouse kicking kinematics is surprising, as any similarities between these traditional martial arts disciplines are likely to represent the fundamental components of this key skill. To date, however, no published studies have reported 3D kinematic data on roundhouse kicking technique across these three styles in highly skilled participants.

Consequently, the purpose of this study was to analyse and compare the 3D kinematics of the kicking leg during roundhouse kicks as performed by high-level practitioners from each of these three traditional martial arts disciplines (Taekwondo, Muay Thai, and Karate). It was hypothesized that key performance indicators (KPI) would exist irrespective of the assumed differences between disciplines. These KPI would include movement characteristics found within each discipline groups kicks (i.e. coordination sequence, rates of joint movement.

## Methods

### Participants

Twenty-four advanced/black belt level Muay Thai, Karate and Taekwondo practitioners (*n* = 8 per discipline) agreed to participate in this study. Inclusion criteria were that participants be no younger than 16 years of age, have a minimum of 5 years of regular training and recognised as being a highly skilled practitioner within their respective discipline/style (i.e. holding a Karate or Taekwondo black belt or a high competitive Muay Thai ranking). Descriptive data for participants are presented in Table 1. Before testing, all participants were informed of the testing procedures and completed an informed consent form. This research was approved by the University of the Sunshine Coast Human Research Ethics Committee (HREC: 90 S/10/2066).

**Table 1:** Descriptive participant data for each martial art group.

Group	Age (yrs)		Height (m)		Mass (kg)		Training Age (yrs)	
	Mean	SD	Mean	SD	Mean	SD	Mean	SD
Muay Thai	22.3	4.1	1.746	0.096	65.6†	8.4	6.4	4.0
Taekwondo	28.6	9.5	1.778	0.05	95.8	13.4	14.8	7.9
Karate	30.3	10.7	1.789	0.137	84.5	20.1	11.4	8.1
Total	27.0	8.9	1.767	0.373	82.0	19.0	10.9	7.5

^†^ Significantly different from Taekwondo

### Data collection

Participants completed a 10 min warm-up including several practice trials before testing. Participants were instructed to produce a number of “quality” kicks against an instrumented target pad using their dominant kicking leg. The quality for any given kick was determined by the subjective assessment of each participant (i.e. did the movement, speed, impact and contact area feel good or bad?). Multiple trials were allowed if participants were unsatisfied with a performance, with the three ‘best’ kicks selected for analysis. The contact area of the kicking leg was defined as the dorsal surface of the foot to the mid-anterior surface of the shin. Each trail began with a chime that signified the start of a 10 s window in which the participant was required to complete a kick. A 60 s rest period between each trial was enforced to reduce the influence of fatigue on results.

**Fig 1:**
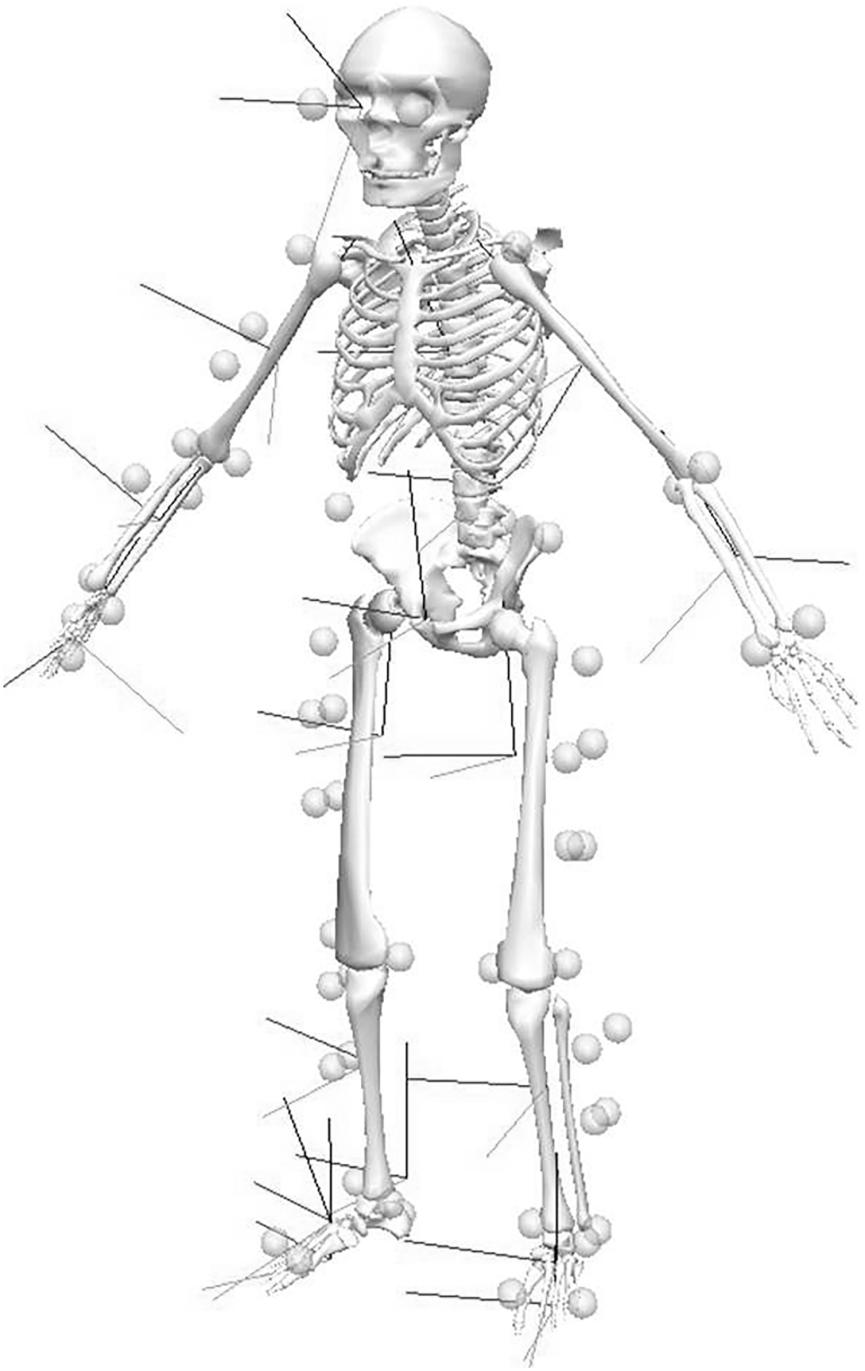
Three-dimensional view of reflective marker placement on participants to track the movement of bodily segments during roundhouse kicking.

Before testing, low mass, retro-reflective markers were attached bilaterally over the anterior and posterior superior iliac spines, greater trochanters, medial and lateral condyles, medial and lateral malleoli and on the lateral distal edge of the 5^th^ metatarsal and the superior surfaces of the 1^st^ metatarsal. Four point marker clusters were positioned on the lateral mid segment aspect on both upper and lower legs (Fig 1).

Kinematic data were then captured using a seven camera Qualisys Motion Capture System (Qualisys AB, Gothenburg, Sweden) sampling at 500 Hz. The target pad was mounted on a metal plate, which was attached to a metal pole via a strain gauge. This configuration was inserted into the adjustable kicking rig. The horizontal midline (across the width) of the target pad was marked using white micropore tape to act as a reference point: this allowed for the adjustment of the height of the target with respect to the participant’s mastoid process level. The position of the strain gauge was orientated to capture horizontal (along the x-axis) impact force data. These data were captured (1000 Hz) with a PowerLab system (PowerLab 8SP, ADInstuments, Inc. USA), and synchronized with the kinematic data via an AD converter (NC/USB/19 Rack, Qualisys AB, Gothenburg, Sweden). Calibration of this system was performed using a series of calibration weights (5kg– 30kg) before each testing sessions.

**Fig 2:**
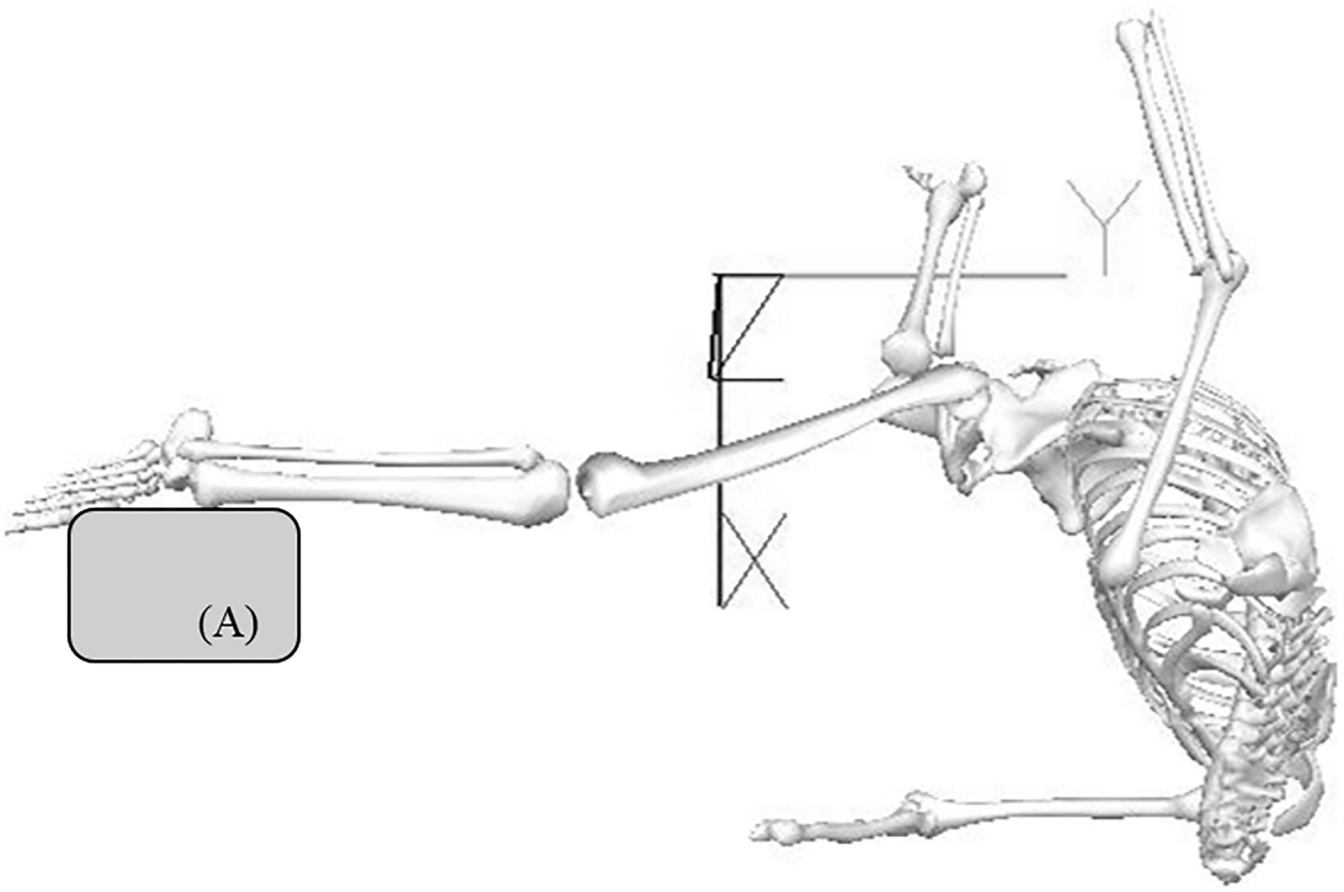
Example of target pad orientation relative to a global coordinate system for a right roundhouse kick. Target Pad (A).

To avoid potential smoothing artefacts influencing data during the Extension phase (particularly around impact) data were filtered using a time-frequency algorithm [28]. The cut-off frequencies during all but the Extension phase were 12 Hz, with the latter filtered at 100 Hz. A 7 segment 3D model of the lower limbs was then created using Visual 3D biomechanical software (Visual3D, C-Motion, Inc. Maryland, USA). This software uses normative segmental inertia data to calculate individual segment centre of mass (COM), which are in turn used to develop total body COM trajectories. A global referencing system was generated with the target pad positioned perpendicular to negative Y-axis (Fig 2). Pelvis kinematics were calculated relative to the global reference system with anterior–posterior tilt, lateral tilt and axial rotations defined using Euler angle calculations of angular rotation about each segment’s X, Y, and Z-axes. Lower limbs were modelled using standard procedures [29] so that flexion, adduction, and medial rotation were defined as positive rotations of the distal segment about the joint’s respective X, Y, and Z-axes. The movement angles were normalized using mean angles from a static capture.

**Fig 3:**
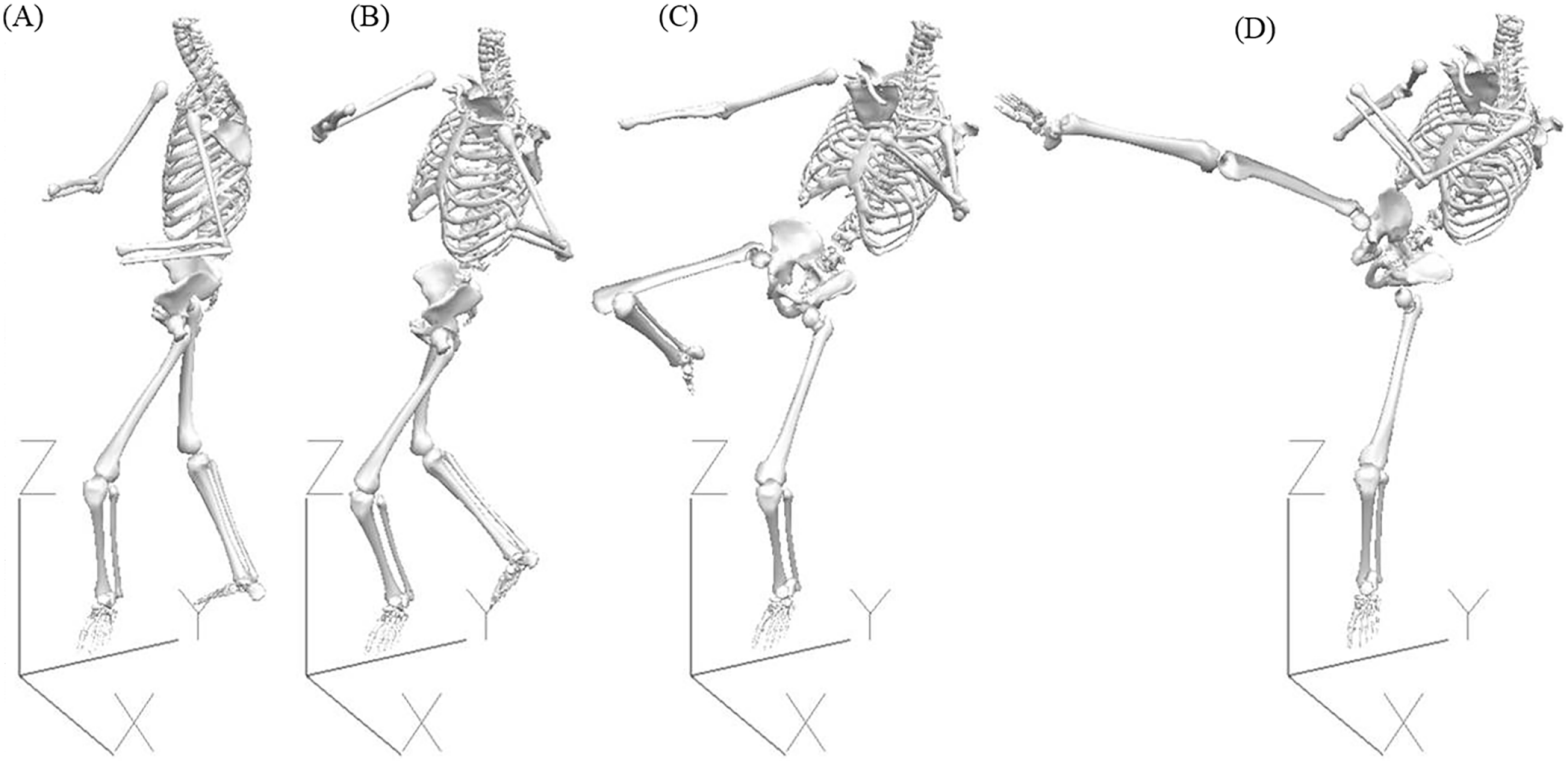
Example of the postures adopted by the participants at the Preparation (A), Chamber (B), Extension (C), and Recoil Phases (D). Figure includes the orientation of the global coordinate system (GCS).

Kicks were broken into four phases (Fig 3) based on previous research in ball sports and martial arts [10,12,25,30,31]. The Preparation Phase was defined as occurring from toe down of the support leg until toe off of the kicking leg. The Chamber Phase started at the end of Preparation Phase and continued until the beginning of knee extension in the kicking leg. The Extension Phase followed and continued until contact with the target pad. The final phase was the Recoil Phase, and it commenced immediately after contact with the kicking pad had ended, and terminated with heel down of the kicking leg. The amount of time from the start of the Preparation Phase to the end of the Recoil Phase was recorded as the Execution time. In addition to these temporal variables standard pelvis and lower limb kinematic variables were developed from previous research on both martial arts kicking [8,9,17,18] and kicking in ball sports [25,27,32–34].

### Statistical analysis

Three-dimensional descriptive statistics were calculated on the pelvis and lower limb kinematic data together with impact kinetics for individuals and each group. The differences between kicking phase durations, joint angular displacements and velocities at key kicking events, and impact kinetic data were determined for the three groups by use of a one-way ANOVA. Scheffe’s Post Hoc procedures were applied to determine whether specific differences in independent variables occurred between disciplines. These data are present at means ±1 standard deviation (SD) with a confidence level of 95%. The relatively small sample sizes meant that Standard Effect (ES) statistics [35] were also used. A relative measure of the differences between groups was reported based on an ES< 0.2 as *negligible*, ES between 0.2 and 0.5 as *small*, 0.5–0.8 as *moderate* and > 0.8 as *large*. Pearson’s product-moment correlations were performed for linear foot velocity at impact against both absolute and relative impact forces with a confidence level of 99.9%. Statistical analysis of the data was performed using the statistics package SPSS for Windows (version 22).

## Results

The Muay Thai group had significantly less body mass than the Taekwondo group, but not compared to the Karate group (Table 1). There were no significant differences in body mass between Karate and Taekwondo. No significant differences existed between the group heights or the average number of years of training.

**Fig 4:**
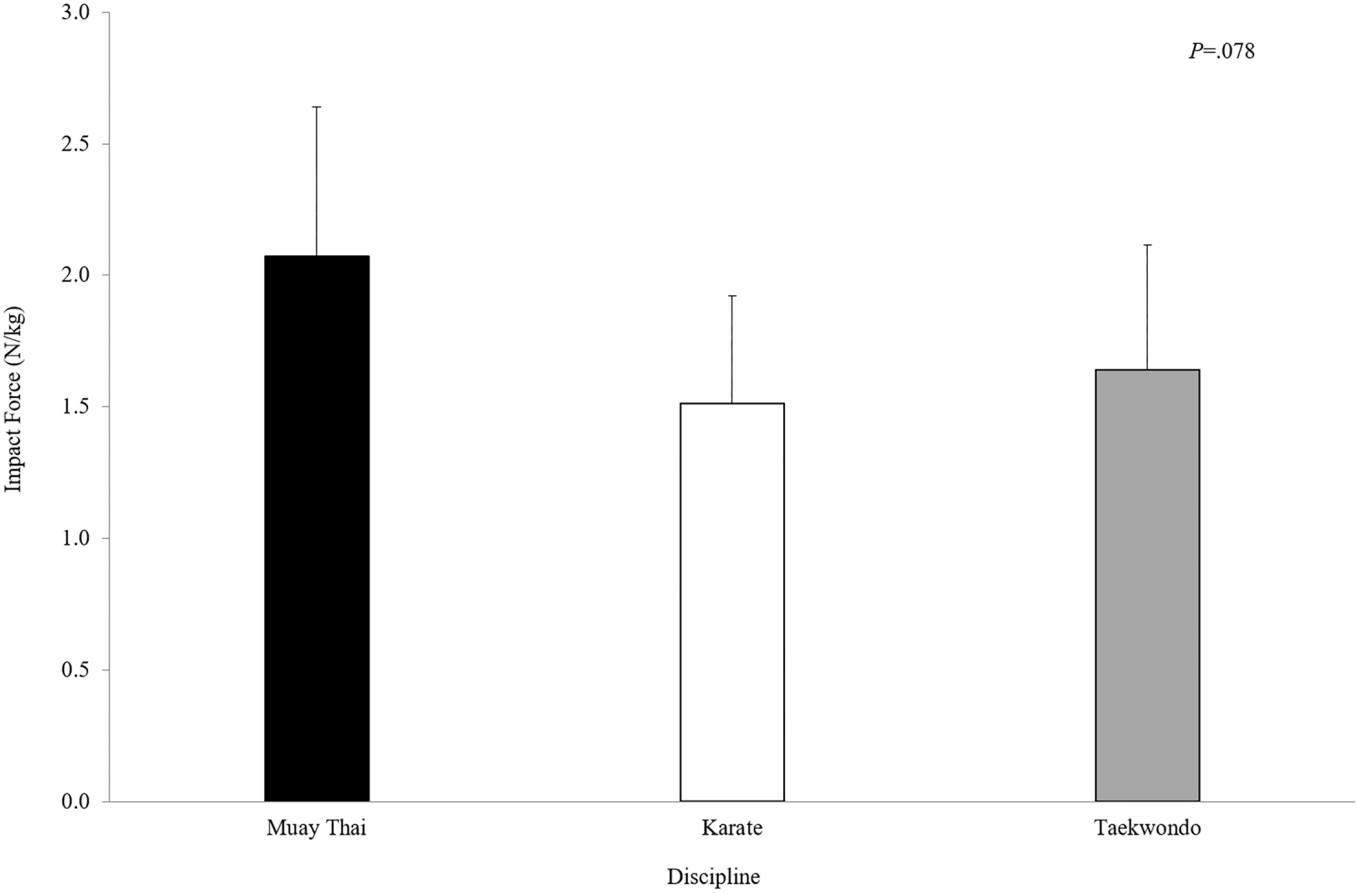
Mean impact forces represented as a function of body weight for Muay Thai (n = 8), Karate (n = 8) and Taekwondo (n = 8) groups (error bars represent +1SD).

Kick execution times and maximum linear foot velocities did not differ significantly between disciplines, although differences were apparent in the linear foot velocities at impact (Table 2). Additionally, the linear foor velocities recorded *weak* to *moderate* correlations with normalised mean impact forces (Muay Thai *r* = 0.63, P = 0.129; Karate *r* = 0.14, *P* = 0.745; Taekwondo *r* = 0.45, *P* = 0.266). Similarly, impact forces did not differ significantly (*P* = 0.281) between the Taekwondo (1547 ± 530 N), Muay Thai (1400 ± 419 N) and Karate (1211 ± 219 N) participants. The normalised mean impact forces (as a function of body mass) showed higher, albeit not significant relative impact force production for the Muay Thai participants (Fig 4) compared to both Karate (*P* = 0.096, ES = 1.13) and Taekwondo (*P* = 0.232, ES = 0.83), with the latter being similar (*P* = 0.873, ES = 0.29). Mean impulse at impact did not differ significantly between disciplines (*P* = 0.367), with values for Karate being *moderately* lower (30.9 ± 6.9 N.s) than both Muay Thai (38.1 ± 12.3 N.s) and Taekwondo (38.8 ± 15.3 N.s).

**Table 2:** Mean, maximum angular velocities and range of motion (ROM) data (± SD) at the pelvis, hip, and knee during Extension phase.

Variable	Muay Thai			Karate			Taekwondo		
	Mean	SD	Range	Mean	SD	Range	Mean	SD	Range
Kick execution time (s)	1.02 ^LK, LT^	0.15	0.41	1.29 ^MT^	0.28	0.76	1.54	0.52	1.70
Max linear foot velocity (m/s)	13.24 ^SK, MT^	2.30	6.61	13.66 ^LT^	1.06	3.31	14.66	1.18	3.99
Linear foot velocity at impact (m/s)	7.22* ^LK, MT^	1.47	4.27	5.57 ^LT^	0.80	2.32	6.36	0.89	2.70
Max knee flexion (deg)	99 ^ST^	11	32	99 ^MT^	6	15	96	2	6
Knee flexion at impact (deg)	43 *^†^^LK, LT^	7	25	15	6	18	16	10	33
Hip flexion at impact (deg)	25 ^MK, ST^	31	80	39	12	36	35	30	85
Hip abduction at impact (deg)	53 ^MK, MT^	3	9	50 ^ST^	7	19	51	4	13
Hip internal rotation at impact (deg)	10 ^ST^	13	38	8 ^MT^	15	45	15	17	53
ROM pelvis posterior tilt (deg)	71 ^MK, ST^	9	27	77 ^ST^	9	22	75	8	20
ROM pelvis lateral tilt (deg)	6 ^SK, ST^	7	18	10	23	75	12	24	68
ROM pelvis forward axial rotation (deg)	92 ^LK, LT^	23	72	114 ^ST^	30	100	122	27	82
Max pelvis posterior tilt velocity (deg/s)	177	27	74	182	31	76	176	48	119
Max pelvis lateral tilt velocity (deg/s)	265 ^SK, ST^	142	435	226	45	141	219	32	99
Max pelvis axial rotation velocity (deg/s)	493 ^SK, ST^	206	686	452	71	204	448	30	91
Max hip flexion velocity (deg/s)	281 ^SK, ST^	135	396	250 ^MT^	72	222	341	162	504
Max hip abduction velocity (deg/s)	-330*	46	118	-262 ^†^^LT^	79	244	-329	45	134
Max hip adduction velocity (deg/s)	12 ^MK, LT^	38	109	48 ^MT^	87	249	95	84	235
Max knee extension velocity (deg/s)	-706 *^†^^LK, LT^	200	556	-947	94	258	-943	106	336
COM anterior displacement (m)	1.40 ^SK, MT^	0.26	0.79	1.26	0.39	1.14	1.25	0.32	0.80
COM lateral displacement (m)	0.37 ^LK, LT^	0.40	1.35	-0.63 ^ST^	0.47	1.51	-0.32	0.81	2.08
COM vertical displacement (m)	1.23 ^LK, LT^	0.16	0.53	0.78 ^MK^	0.24	0.69	0.93	0.19	0.65

* Significantly different than the Karate (*P* < .05)

^†^ Significantly different than Taekwondo (*P* < .05)

^SK^ s*mall* ES with Karate (*d = 0*.2–.5) ^ST^ *small* ES with Taekwondo (*d* = 0.2–.5) ^MK^ *moderate* ES with Karate (*d* = 0.5–.8) ^MT^ *moderate* ES with Taekwondo (*d* = 0.5–.8) ^LK^ *large* ES with Karate (*d*>.8) ^LT^ *large* ES with Taekwondo (*d*>.8).

**Fig 5:**
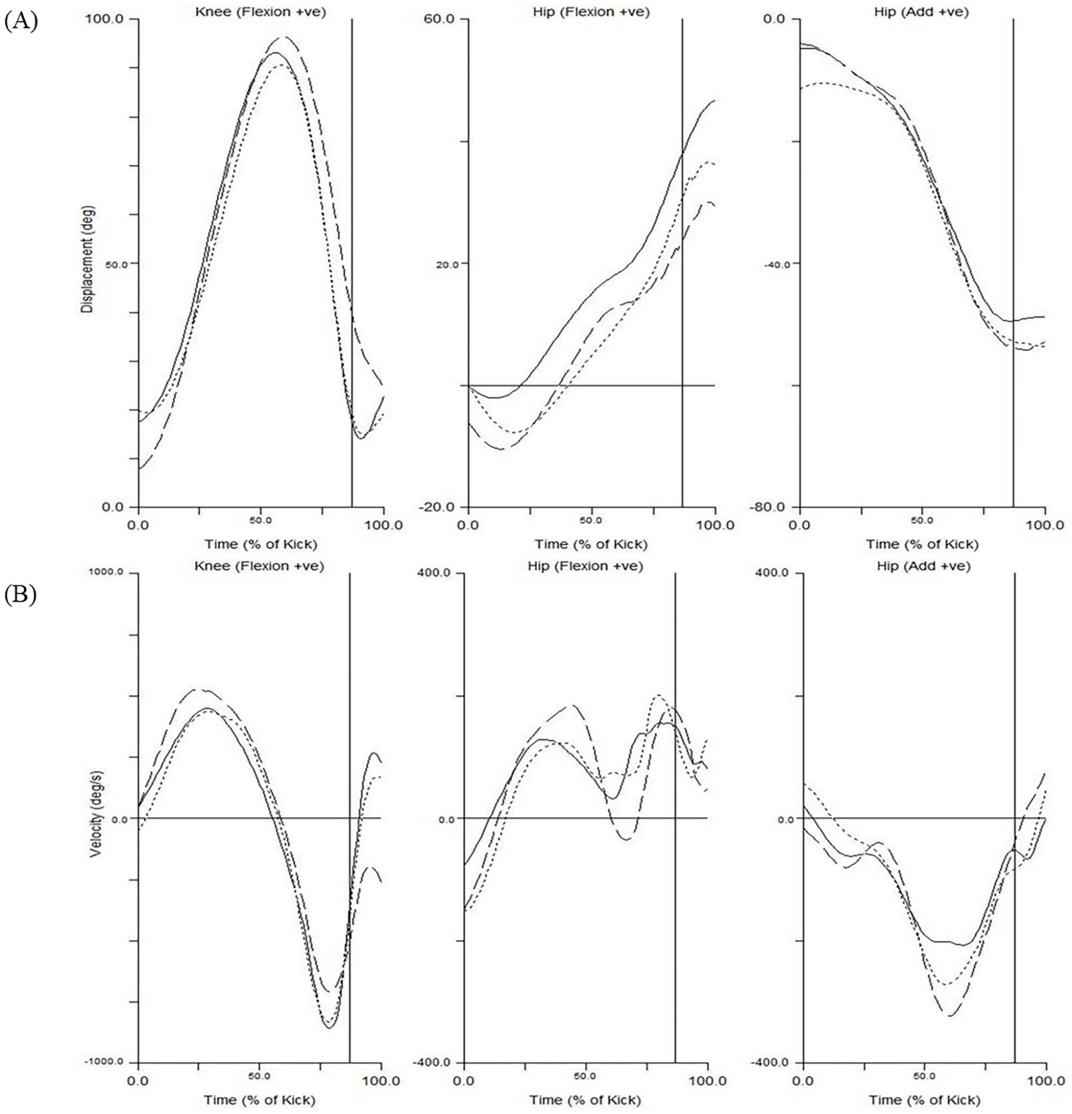
Mean kicking leg 3D hip and knee joint displacement and velocity data for Muay Thai, Karate, and Taekwondo groups. Displacement (A) and velocity (B). Muay Thai (dashed), Karate (solid) and Taekwondo (dotted line). Data are time normalized from the start of the Chamber Phase (0%) until 0.1 s after impact with the kicking pad (100%). Impact with the kicking pad is shown as a vertical line.

The relative durations (as a % of execution time) for each of the four kicking phases were similar between disciplines. The Recoil phase (69%) constituted the majority of the kick followed by the Chamber (14%), Extension (11%) and Preparation (6%) phases. ANOVA testing showed significant differences recorded for mean execution times (*P* = 0.028) between Muay Thai and Taekwondo, with no significant differences between these two disciplines and Karate. A significant difference was found in the Extension phase durations between Muay Thai and Karate (*P* = 0.01) while no difference existed between Karate and Taekwondo or Taekwondo and Muay Thai. *Small* non- significant variations were observed in the Preparation (Muay Thai 0.074 ± 0.018 s, Karate 0.087 ± 0.073 s and Taekwondo 0.080 ± 0.054 s) and Recoil phases (Muay Thai 0.65 ± 0.173 s, Karate 0.85 ± 0.307 s and Taekwondo 1.14 ± 0.534 s). There were *large* differences between Muay Thai and the other disciplines when assessing joint orientations at Impact and maximum angular velocities of the pelvis, hip joint, and knee joint during the Extension Phase (Table 2). All groups exhibited similar values for maximum pelvic posterior tilt, lateral tilt and forward axial rotation velocities, with the larger of these velocities being the rate of forward axial rotation (Table 2). Additionally, maximum hip adduction velocities were considerably slower than those recorded for hip abduction. Time-normalized kinematic data shows that peak pelvic axial rotation velocity corresponded to a marked reduction in hip flexion velocity with the hip undergoing extension (relative to the pelvis) in all Muay Thai and Taekwondo kicks and half of the Karate kicks (Fig 5). The point of maximum knee extension velocity occurred before contact with the kicking pad.

The vertical displacement of the COM during the kick was significantly greater for the Muay Thai group than that for Karate (*P* = 0.001) and Taekwondo (*P* = 0.02) groups (Table 2). There were no significant differences in anterior displacement of COM where values were almost identical for Karate and Taekwondo, while Muay Thai had *small* to *moderate* positive differences. There was no significance between COM lateral displacements for Muay Thai, Karate, and Taekwondo. Linear foot velocity at impact recorded significant moderate correlations with both absolute (*r* = 0.66, *P* = 0.001) and relative (*r* = 0.60, *P* = 0.001) impact forces. None of the other measured kinematic variables recorded significant correlations with mean impact forces.

## Discussion

Previous studies on roundhouse kicking report on the kinematics and kinetics of a single martial arts discipline [5,7,9,10,20,23,24]. This novel study analysed and compared the 3D kinematics of the kicking leg during roundhouse kicks being performed by highly qualified practitioners from three traditional martial arts disciplines. It was hypothesised that key performance indicators would be those characteristics found across each discipline. We assessed known key determinants of performance [5,7,9,10,20,23,24] and included 3D analyses of the involvement of the pelvis during this complex multi-planar skill. Our results suggest that a typical roundhouse kick is characterised by a horizontal and vertical shift of the COM towards the target, which is coupled with a rapid forward pelvic axial rotation, hip abduction, hip flexion, and knee extension.

Kick execution times of the three groups in the current study are similar to those reported for Karate [19], although slightly slower than those presented in studies on Taekwondo [5,10,24,36]. This disparity is likely a result of the different definitions of key events and kick phases used during previous research studies, with this lack of standardisation being acknowledged as an issue in this field [5]. Kicking execution time [5,7,24,25] and kicking impact force [5,11] have been identified as crucial aspects of Taekwondo performance. For example, Falco et al. [5] indicated that expert martial arts competitors produce greater impact forces and have faster skill execution times than novice competitors (0.254 s versus 0.317 s). However, the static nature of the test procedures in the current study may have resulted in an overemphasis on the Recoil phase by participants through a delay in returning the kicking leg to the ground. This delay is likely a consequence of the participants’ enthusiasm for generating large impact forces in the absence of the risks usually associated with a fight in competition. For example, in competitive sparring/fighting, there is a need to maintain balance and limit openings for attack or counter attack is paramount and so it is essential that successful competitors promptly return their kicking leg to the ground. Importantly, results show that the relative time of each kicking phase is the same regardless of discipline.

Results indicate that despite the roundhouse kicking kinematics being predominately similar across all three disciplines, significant and *small* to *large* differences were evident in several areas (Table 2). Subtle differences such as these may represent the differing principles of the three disciplines (i.e. quick controlled snap-like Kick, Karate; vs. large windup based kicks, Taekwondo; vs. kicks that angle the shin down during impact, Muay Thai). Although these different *ideals* of kicking are subjective, and evidence is anecdotal at best, participant’s attempts to fulfil these *ideals* may help explain the presence of slight differences in variables such as hip abduction and hip flexion velocities. The rates of knee extension in the Muay Thai were slower than both Karate and Taekwondo, which would explain why the Muay Thai group had a substantially more flexed knee at impact. Linear foot velocity at impact and knee extension velocity have been shown to be key determinants of ball velocity in other kicking sports [25–28,30,37,38].

The relative durations of the kicking phases were similar across all groups regardless of kick Execution time. Accordingly, differences in Extension phase durations for the Muay Thai group were, therefore, likely a function of the shorter kicking Execution time. This may be due to the cumulative effect of the faster hip abduction, lateral pelvic tilt and axial rotation velocities demonstrated by this group (i.e. *small* to *medium* differences that were not significant). The accumulation of these slight differences resulted in the Muay Thai participants initiating their kicks closer to the target, reducing the distance travelled by the kicking leg and shortening Execution time. These slight differences in proximal leg velocities may also explain how Muay Thai participants were able to produce higher foot velocities at impact despite having slower knee extension velocities. However, further research on their use of pelvic rotations is clearly required before definitive conclusions can be made.

The data presented here confirms that linear foot velocities, as well as the anterior pelvic tilt and axial rotation velocities, have a pivotal role when it comes to roundhouse kick impact force production. Our maximum linear foot velocities values were similar to those reported in studies involving similar level participants [8,15]. However, these relationships did not predict impact force outcomes between differing groups. This is evident when comparing the normalised mean impact forces with the corresponding maximum linear foot velocity, anterior pelvic tilt and the forward pelvic axial rotation velocities (Table 2). Interestingly the linear foot velocities at impact presented in the current study were 41%–54% of the maximum linear foot velocities recorded during the Extension phase. This change is also reflected in the large deceleration in knee extension velocities that occurred prior to impact with the pad. Similar reductions have been reported during Taekwondo roundhouse kicks at a small hand held target [8]. It is probable that these reductions are a function of the testing protocols which required participants to kick a relatively immovable object.

While significant differences exist in the maximum knee extension velocities between the groups. It should be noted that maximum knee flexion and extension velocities in the present study were consistent with values from Karate [18], and Taekwondo kicks [36], but slower than those presented by O’Sullivan et al. [9]. While peak knee extension velocity has an important role in determining linear foot velocity at impact, clear technique differences exist between these three martial arts disciplines. For example, our data indicates that the typical Karate and Taekwondo *snap-like* kick is based heavily on rapid knee extension, while Muay Thai uses a technique that places greater emphasise on COM movements to develop high impact forces. Importantly, the peak knee extension velocities reported here are potentially more accurate than previous research, as most martial arts research is based on kinematic sampling rates of less than 200 Hz and/or 2D data collection procedures, both of which are likely to underestimate these values [28]. Our knee extension velocities are considerably slower than those reported previously for both punt kicking [25,27] and soccer kicking [28,37,38]. In particular, the rapid deceleration in knee extension velocity immediately prior to impact in our study is not representative of research on ball kicking where peak knee extension velocities occur either at or immediately after ball impact [28]. It is likely that our data are a function of the test protocols that required the participants to strike a pad attached to an immovable frame that was positioned at head height. Accordingly, the kicking plane is likely to result in reduced linear foot velocities near impact, but more importantly, participants decelerated their foot immediately prior to impact to prevent injury. Clearly, further research is required that explores whether this deceleration is present when striking lower mass and or moving targets.

All groups tended to shift the COM toward the target during the Extension phase with a large emphasis on the vertical shift of COM. This greater vertical shift is a function of target height and is consistent with previous research [10]. The movement of COM towards the target is common among kicking skills and is the means of transferring the body’s momentum to the target [6,37–39]. However, the greater vertical shift in the COM present in the Muay Thai participants (between 0.3 m and 0.45 m) is a novel finding of this study and suggests a clear difference in “target approach” between this discipline and both Taekwondo and Karate.

The rapid 3D pelvic rotational velocities reported in the current study suggest that these movements are important components of this skill. Of these values, the pelvic axial rotation velocities were around 2.5 times faster than the posterior tilt velocities. Kim et al. [12] is the only study to have reported the complex 3D interactions between the kicking leg and the pelvis during roundhouse kicking. These researchers indicated that during the roundhouse kick the pelvis tilted posteriorly and laterally through approximately 60 deg, and rotated through approximately 130 deg. Research on other kicking activities has suggested that the posterior tilting of the pelvis prior to the forward leg swing creates a tension arc across the front of the hip joint [25–27,38]. In addition, a study on rugby punt kicking suggested that highly proficient kickers combine axial pelvic rotation with the posterior pelvic tilting to increase the magnitude of the tension arc across the hip [26]. The highly skilled martial artists in the current project also appeared to use this technique to improve their kicking velocity.

Maximum rates of hip flexion reported in the current study were smaller than those described previously for both Karate and Taekwondo [9,18]. However, this was likely a function of the fact that other researchers in this field typically represent hip flexion/extension as the orientation of the thigh with respect to the trunk as opposed to pelvis [9,11,19,20,31]. This practice will overestimate the hip flexion velocities, particularly as the trunk is flexing forward at this stage of the kick. The maximum hip abduction velocities in the present study were substantially larger than that of hip adduction, but this was likely a function of target height and may not be representative of roundhouse kicks to the chest or thigh [9,10,24]. This indicates that comparisons between the more sagittal plane types of kicking seen in ball sports with martial arts kicks traveling in the transverse plane are limiting and misleading. Further data collection during combat situation would indicate whether this is a functional difference caused by the movement type and/or target height or an artefact caused by the data collection procedures.

The mean impact forces recorded in the current study are less than reported in previous research on Taekwondo roundhouse kicks [5,9,11]. Interestingly, the significantly lighter Muay Thai group (i.e. lower body mass) was able to produce impact forces similar to that of the heavier participants from Karate and Taekwondo, supporting research reporting that body mass does not influence impact force in expert competitors [5]. However, our impact force data are considerably greater than the 292 N reported by Pearson [15]. These differences highlight the lack of consistency in assessing these data across the literature making cross-study comparisons extremely difficult [5,9]. Another factor that is likely to influence the relative differences between studies is highlighted by the relatively large SD values in impact forces for each group (between 20% and 30%). Such large inter-individual values indicate that this is not a reliable performance measure, and so care should be taken to avoid over-interpreting results. When combined with results regarding the movements of the COM, the additional force in Muay Thai kicks was influenced by the significantly larger vertical displacement of COM during the Extension phase.

The selection criteria for this study limited involvement to high-level martial arts practitioners, and so results may not be representative of the general population of participants in these sports. However, a strength of this study is the use of high calibre athletes with all participants having a well-developed and refined technical ability (N.B.: the “grading” processes entrenched in these martial arts are all based on expert assessment of technical skill). This inclusion criterion had the effect of limiting sample size, and so some care should be taken before attempting to generalise these results across wider populations. Another confounding aspect of this research was the large inter-individual differences present in most variables across disciplines. In some cases, the SD values were greater than 50% of the group means. Although intra-individual differences were smaller, the large SD were unexpected because it was assumed that highly skilled participants would reproduce relatively similar movement patterns within the respective disciplines. These findings show that within the rigid movement constraints imposed by the traditional martial arts disciplines, that there exist subtle (*small—large*) movement differences between each of these high-level participants. This ‘self-organizing’ strategy is consistent with the degeneracy concept, which suggests that high-level performers often incorporate subtle differences in movement patterns to achieve a set outcome [40]. However, a limitation of this and other studies is the use of a static and restrained target. Accordingly, caution should be taken before attempting to generalize these results to include competition or live sparring conditions.

In conclusion, our results indicate that several fundamental movement patterns are common to the roundhouse kicking techniques adopted by our high-performance practitioners from each of the Muay Thai, Karate, and Taekwondo disciplines. Effective roundhouse kicking performance in our population was characterized by a combination of rapid pelvic axial rotation, hip abduction, hip flexion and knee extension velocities, combined with rapid movements of the COM towards the target. While subtle differences exist for each of these key variables across our sample, we suggest that these elements have the potential to constitute key performance indicators for this fundamental martial arts skill.
